# Epidemiological Analysis of Coronavirus Disease 2019 (COVID-19) in 2 Cities in China Based on Public Data

**DOI:** 10.1017/dmp.2020.401

**Published:** 2020-10-26

**Authors:** Ning Zhou, Xiaomeng Zhang, Ying Zhang, Lu Gao, Penghui Zhou, Hui Liu

**Affiliations:** 1 Tianjin Center for Disease Control and Prevention, Tianjin, China; 2 Tianjin Center for Tuberculosis Control, Tianjin, China

**Keywords:** coronavirus disease 2019 (COVID-19), epidemiology, public data

## Abstract

Based on the public data from the health departments of Tianjin and Shenzhen, we conducted a comparative analysis of the coronavirus disease 2019 (COVID-19) epidemic situation between these 2 cities. The aim of this study was to evaluate the role of public data in epidemic prevention and control of COVID-19, providing a scientific advice for the subsequent mitigation and containment of COVID-19 prevalence.

On December 29, 2019, a hospital in Wuhan reported a cluster of unknown severe pneumonia cases for the first time, which was timely notified to the World Health Organization (WHO) after verification by the Chinese government.^1^ On January 8, 2020, the pathogen of this pneumonia was finally confirmed as severe acute respiratory syndrome coronavirus 2 (SARS-CoV-2). On January 30, WHO declared the novel coronavirus pneumonia outbreak as a public health emergency of international concern.^2^

In the nearly 5 mo of global fights against the coronavirus disease 2019 (COVID-19), it displayed different trends of the COVID-19 epidemic with different prevention and control measures. For this novel disease, finding a rapid and effective way to control it is a comprehensive analysis of the information of each confirmed case. Therefore, WHO provided a list of basic relevant variables in a standard format to collect global COVID-19 case information.^3,4^

With the outbreak of COVID-19, nearly 100 cities in China released epidemiological information of confirmed COVID-19 cases in time.^5^ In this study, we selected 2 cities, Tianjin and Shenzhen, for comparative analysis. Three main reasons for choosing these 2 cities were as follows. First, Tianjin and Shenzhen both released COVID-19 related information timely and continuously. Second, these 2 cities are regions with similar economic levels, belonging to the Beijing-Tianjin-Hebei synergetic zone and the Zhujiang-Xijiang economic belt, respectively. Third, these 2 cities have large populations (> 10 million), which can provide sufficient data to analyze.^6^ In this study, based on the comparative results of the public data from these 2 cities, we found that the open data played an important role in epidemic prevention and control, providing a scientific advice for the subsequent mitigation and containment of COVID-19 prevalence.

## Methods

### Sources of Data

The epidemic data were collected from the confirmed COVID-19 cases published daily on the official websites of Tianjin and Shenzhen Health Committee. Data were collected before February 29, 2020. During this period, 136 and 417 confirmed cases were collected in Tianjin and Shenzhen, respectively.

We made a specific list of variables for this study according to the variable list of confirmed COVID-19 cases released by the WHO. The collected information contained age, gender, current address, disease exposure source, onset date, treatment date, confirmed date, hospital date, discharge date, clinical symptoms, clinical classification, death date, the relationship between cases, the virus nucleic acid testing number, and the epidemiology track after disease. Therefore, a database of confirmed cases from Tianjin and Shenzhen was established.

### Diagnostic Criteria

All the cases released by Tianjin and Shenzhen were regarded as confirmed cases. According to the National Guidelines in Diagnosis and Treatment Scheme for COVID-19 (Version 1-7), the clinical severity of COVID-19 cases was categorized as mild, moderate, severe, and critical. Patients were defined as having the source of exposure if the epidemiological history of the case were published within 14 d before symptom onset, and having the clinical symptoms if they published any of the clinical manifestations. Patients who published epidemiological track after symptom onset were defined as having the epidemiology track after disease. In addition, each patient requires 1-2 nucleic acid tests, but in our study, the number of virus nucleic acid detection beyond the regulations is considered as the announcement of the number of virus nucleic acid detection.

### Statistical Analysis

All confirmed cases were statistically analyzed by SPSS 23.0 software (SPSS, Inc., Chicago, IL). Categorized variables were expressed by numbers and frequencies, continuous variables were presented as means or medians. The interval of time from onset to treatment date was used to evaluate the effect of public information on epidemic control.

## Results

### Baseline Data of Confirmed COVID-19 Cases in Tianjin and Shenzhen

Clinical information of case epidemiology published by Tianjin and Shenzhen were listed in Table 1. The age, gender, and current address of the cases in the 2 cities were all released (100.00%). For the exposure source, 126 cases (92.65%) in Tianjin and 403 cases (96.64%) in Shenzhen were released publicly. For onset date, 87 cases (63.97%) were publicly released in Tianjin and 415 cases (99.52%) in Shenzhen. For treatment date, 87 (71.32%) and 417 (100.00%) cases were released publicly in Tianjin and Shenzhen, respectively. For confirmed date, 136 cases (100.00%) in Tianjin and no cases in Shenzhen were released publicly. For discharge date, no cases in Tianjin and 262 cases (62.83%) in Shenzhen were released publicly. For clinical symptoms, there were, respectively, 69 (50.74%) and 12 cases (2.88%) in Tianjin and Shenzhen were released publicly. For clinical classification, 109 cases (80.15%) in Tianjin and 18 cases (4.32%) in Shenzhen were publicly released. For the epidemiology track after disease, 113 cases (83.09%) in Tianjin and 50 cases (11.99%) in Shenzhen were released publicly. For the number of nucleic acid tests, 21 cases (15.44%) in Tianjin and no cases in Shenzhen were released publicly.

**Table 1.** Comparison of the baseline data between confirmed cases in Tianjin and Shenzhen

Baseline Variable	Tianjin (*n* = 136)		Shenzhen (*n* = 417)	
	Released	No. of Cases (%)	Released	No. of Cases (%)
Age	Yes	136 (100.00%)	Yes	417 (100.00%)
Gender	Yes	136 (100.00%)	Yes	417 (100.00%)
Current address	Yes	136 (100.00%)	Yes	417 (100.00%)
Exposure source	Yes	126 (92.65%)	Yes	403 (96.64%)
Onset date	Yes	87 (63.97%)	Yes	415 (99.52%)
Treatment date	Yes	97 (71.32%)	Yes	417 (100.00%)
Confirmed date	Yes	136 (100.00%)	No	0 (0.00%)
Discharge date	No	0 (0.00%)	Yes	262 (62.83%)
Clinical symptoms	Yes	69 (50.74%)	Yes	12 (2.88%)
Clinical classification	Yes	109 (80.15%)	Yes	18 (4.32%)
Epidemiology track after disease	Yes	113 (83.09%)	Yes	50 (11.99%)
Virus nucleic acid testing number^a^	Yes	21 (15.44%)	No	0 (0.00%)

a Specific information not recommend by WHO.

### Characteristic of the Data Published by the 2 Cities

**Figure 1.**
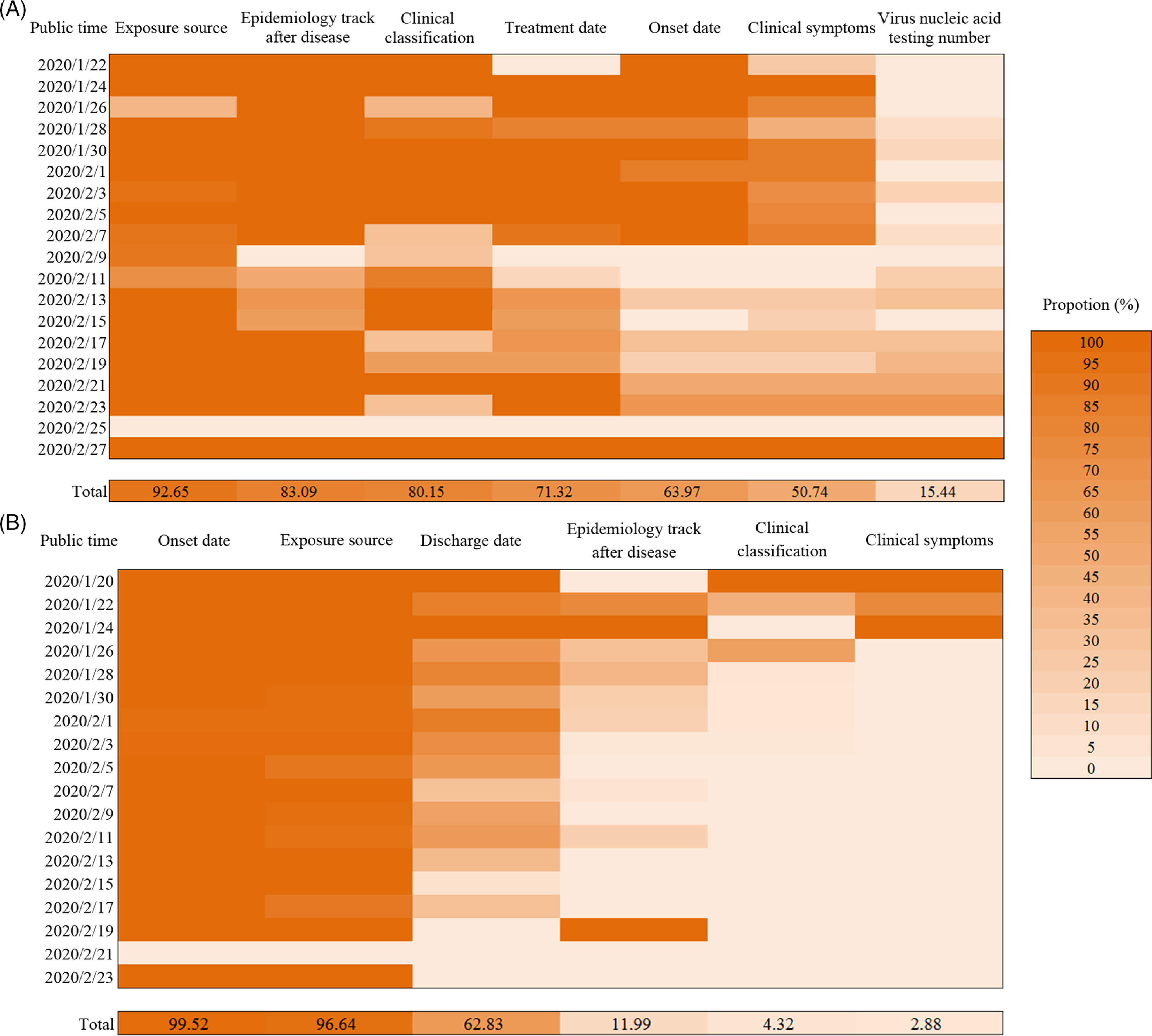
Heatmaps of the diagnoses number of case information variables in different time intervals in Tianjin (A) and Shenzhen (B).

In the data published in Tianjin, with the progress of the epidemic, the published proportion of treatment date, onset date, and clinical symptoms decreased gradually to 71.32%, 63.97%, and 50.74%, respectively (Figure 1 **A**). Meanwhile, the published proportion of the virus nucleic acid testing number increased gradually (Figure 1A). In the data published of Shenzhen, the proportion of epidemiology track after disease, clinical classification, and clinical symptoms in the published cases decreased with the progress of the epidemic situation, which were 11.99%, 4.32%, and 2.88%, respectively (Figure 1B). As of February 26, a total of 262 (62.83%) cases discharged were reported (Figure 1B), and the Shenzhen municipal government has stopped releasing information on discharged cases since then.

### Impact of Public Information on Epidemic Control

**Figure 2.**
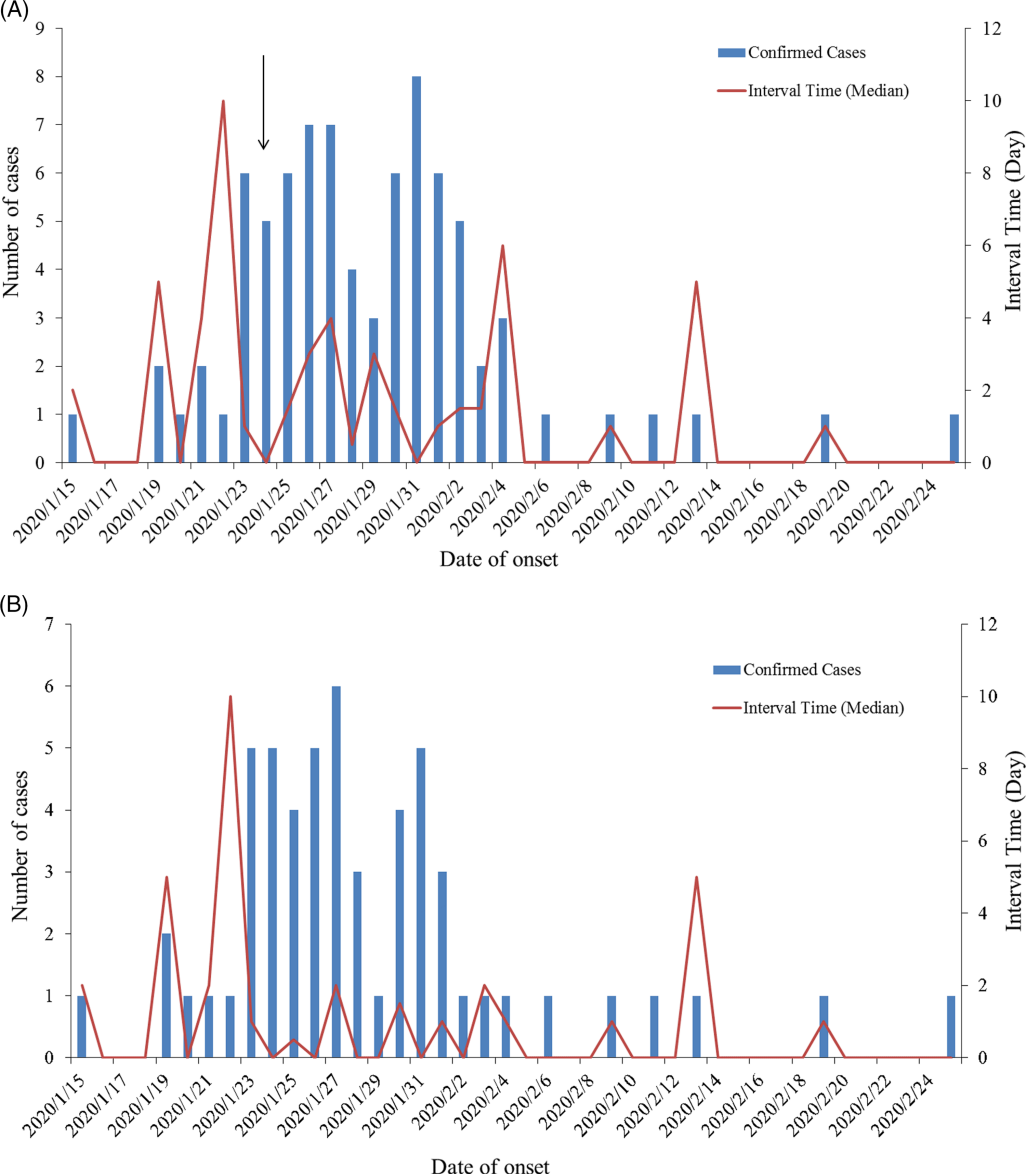
The distribution of COVID-19 onset time and interval time from onset to diagnosis. (A) The onset and interval time in Tianjin. (B) The onset and interval time in Tianjin after eliminating the cluster cases.

Afterward, 81 and 415 cases in Tianjin and Shenzhen with detailed time of onset and diagnosis were selected to conduct a comparative analysis. Figure 2 **A** showed the first peak on January 26 and 27 in Tianjin, and the second peak reappeared after a slight decrease, with the most confirmed cases on January 31 (Figure 2A). The results of statistical analysis of the interval time from onset to diagnosis revealed that there was a slight decline of the interval time (arrow in Figure 2A), but not obviously. Because a cluster event occurred in the shopping mall involved many cases, these cases were eliminated for reasonable analysis and the number of confirmed cases dropped on January 31, displaying only 1 peak on January 26 and 27; the interval time was significantly reduced as well (Figure 2B).

**Figure 3.**
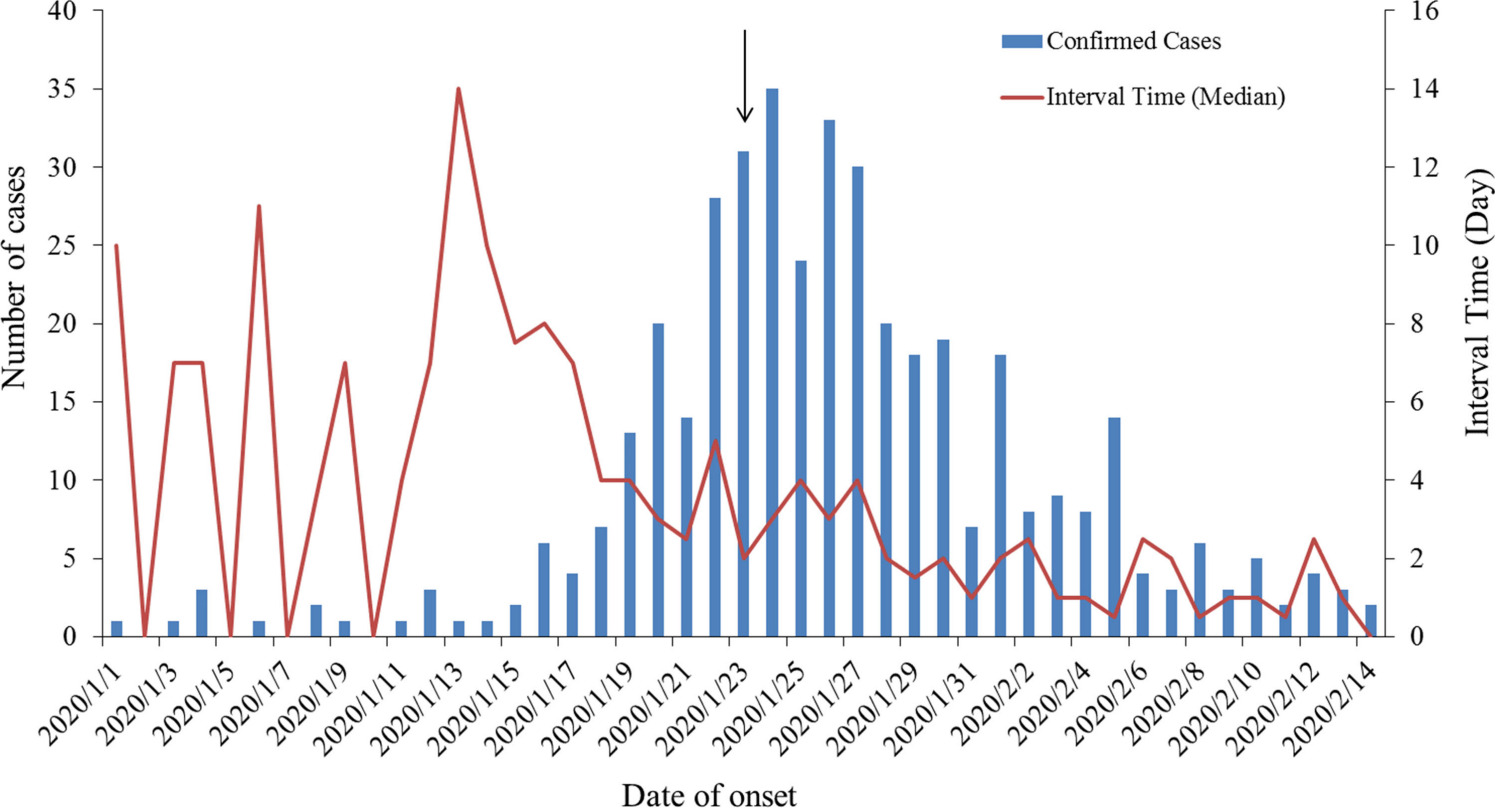
The distribution of COVID-19 onset time and interval time from onset to diagnosis in Shenzhen.

In Shenzhen, there was only 1 peak that occurred from January 24 to 26, and after that, the number of daily confirmed cases decreased (Figure 3). The interval time before January 23 varies a lot, and the median interval time were longer. Since then, Shenzhen activated the highest level of emergency response, and the range of interval time variation gradually narrowed, accompanied by the shorter median interval time (Figure 3).

## Discussion

COVID-19 is the most extensive global pandemic to afflict humanity in the past decades. It is a serious crisis and a daunting challenge for the whole world. In the face of the unprecedented, sudden, and violent outbreak of disease, information disclosure is the best vaccine without special drugs. Shenzhen and Tianjin, 2 large cities in China, 1 south and 1 north, can timely disclose and publish case data, including: age, gender, current address, exposure source, onset date, treatment date, confirmed date, discharge date, clinical symptoms, clinical classification, epidemiology track after disease, and virus nucleic acid testing number. The governments of the 2 cities are in the forefront of comprehensive evaluation of the COVID-19 epidemic information.^5^ The public has real-time understanding of the cases in these 2 cities,^7,8^ which has also triggered a lot of public discussion and private analysis.^9^ Through this information, the public can quickly predict whether they are at risk of infection, and can make timely preventive actions.^10^

The current study found that the completeness of the information released by the 2 cities in order of diagnosis was as follows: basic information (age, gender, current address) was better than exposure information (exposure source, onset date), than clinical diagnosis and treatment information (treatment date, confirmed date, discharge date, clinical symptoms, clinical classification, epidemiology track after disease, and virus nucleic acid testing number). This was also a common rule for the release of case information in other regions. The reason might be that: (1) the basic information of the case was more convenient to obtain; (2) patients might have recall bias or conceal contact history when answering questions from epidemiological investigators; and (3) the information released by the government was usually just after diagnosis, and the follow-up treatment would no longer be collected, resulting in most missing clinical diagnosis and treatment information. Therefore, the lack of information on the incubation period, symptoms at onset, clinical characteristics, and outcomes of the 2 cities resulted in limited role in the analysis of the clinical characteristics of new infectious diseases and the risk assessment of medical resource occupation.

The continuity and stability of the case information variables released by Shenzhen were better than those of Tianjin, while the comprehensiveness was slightly better of Tianjin than that of Shenzhen. The 2 cities have separate emphasis on the release of case information. Tianjin added nucleic acid detection information to the later published cases, indicating that the instability of information is bias caused by the influence of hotspots of public concern. Shenzhen paid more attention to the release of the date of onset and treatment, considering that the time interval between onset and consultation is an important indicator for evaluating the risk of epidemic spread.^11^ On the whole, the continuity and stability of information variables were affected by multiple factors, such as public attention, scientific prevention, and control. However, the public demand for epidemic data has not been fully satisfied by the data supply. From the current situation of uneven release levels and lack of standardization, China still needs to further improve relevant laws and regulations, formulates more specific and operable implementation rules and data standards, so as to make the epidemic data opening work more legal, compliant, uniform, and orderly.

Under the situation of a similar population, the number of confirmed cases and incidence rate in Shenzhen is significantly higher than that in Tianjin. There may be 2 reasons for this finding: first, there is a large gap between the immigrant population and permanent residents in the 2 cities. The proportion of immigrant population in Shenzhen is 65%, and 31% in Tianjin. Second, there is a big gap between the floating populations of Hubei provinces (the COVID epidemic outbreak center) in the 2 cities. According to the big data of Tianyan on Baidu map, during the period from January 1 to 20, 2020, the daily emigrated population in Hubei province with Shenzhen as the destination was 1.73-2.92%, and that of Tianjin was 0.32-0.64%. To sum up, the incidence rate of Shenzhen was significantly higher than that of Tianjin in a similar time period. This may be the main reason for the 2 cities with a larger gap between local and imported cases.

The first cases were reported on January 20 and 21, and the first-level public health incident response were launched on January 23 and 24 in Shenzhen and Tianjin, respectively. The time interval curve from the onset to the time of medical treatment in 2 cities showed the same trend as SARS in 2003.^12^ It took more than 40 d from the onset of the first case to the first time that there were no new cases in the region, which was consistent with the model prediction carried out by Sheng et al.^13^ It has been reported that a 1-d delay in implementing social distancing resulted in a containment delay of 2.41 (95% CI, 0.97-3.86) d.^14^ Although at the early stage of the epidemic, due to the lack of awareness of the symptoms, most of the infected people chose to take medicine on their own, which caused the time interval between the onset and the visit to the clinic of the case uneven. However, due to the early response of the 2 cities, and with the effect of prevention and control measures, the range of average time interval between the onset and treatment was greatly reduced, and the epidemic basically subsided by the end of February.

At the same time, this study also has many limitations and shortcomings. The current study only relies on information published by the government; hence, there will be some bias from the actual epidemic situation.^15,16^ Whether there is a correlation between the severity of the epidemic situation and the level of the epidemic data release still needs further research. In addition, how to effectively control the disease and improve self-protection of hospital staff in the early stage of the epidemic, when the disease is not fully understood and when medical resources are not suitable, should be the focus of future public health emergencies for unknown reasons.

In conclusion, our research proposed a new strategy that, based on public information sources, some basic epidemiological analysis could be conducted on the local epidemic. The epidemiological characteristics and sources of Tianjin and Shenzhen had lots of both similarities and differences. It has certain guiding significance for prevention and control of epidemic situations in different cities.
